# The efficacy of high-throughput sequencing and target enrichment on charred archaeobotanical remains

**DOI:** 10.1038/srep37347

**Published:** 2016-11-24

**Authors:** H. M. Nistelberger, O. Smith, N. Wales, B. Star, S. Boessenkool

**Affiliations:** 1 Centre for Ecological and Evolutionary Synthesis, Department of Biosciences, University of Oslo, P.O. Box 1066, Blindern, Oslo, NO-0316, Norway; 2 School of Life Sciences, Gibbet Hill Campus, University of Warwick, Coventry CV4 7AL, United Kingdom; 3 Centre for GeoGenetics, Natural History Museum of Denmark, University of Copenhagen, Øster Voldgade 5-7, 1350 Copenhagen, Denmark

## Abstract

The majority of archaeological plant material is preserved in a charred state. Obtaining reliable ancient DNA data from these remains has presented challenges due to high rates of nucleotide damage, short DNA fragment lengths, low endogenous DNA content and the potential for modern contamination. It has been suggested that high-throughput sequencing (HTS) technologies coupled with DNA enrichment techniques may overcome some of these limitations. Here we report the findings of HTS and target enrichment on four important archaeological crops (barley, grape, maize and rice) performed in three different laboratories, presenting the largest HTS assessment of charred archaeobotanical specimens to date. Rigorous analysis of our data – excluding false-positives due to background contamination or incorrect index assignments – indicated a lack of endogenous DNA in nearly all samples, except for one lightly-charred maize cob. Even with target enrichment, this sample failed to yield adequate data required to address fundamental questions in archaeology and biology. We further reanalysed part of an existing dataset on charred plant material, and found all purported endogenous DNA sequences were likely to be spurious. We suggest these technologies are not suitable for use with charred archaeobotanicals and urge great caution when interpreting data obtained by HTS of these remains.

Advances in DNA extraction methodology and sequencing technology have allowed for the field of plant archaeogenetics – DNA analysis of archaeological plant remains – to flourish over the last decade^1^,^2^. This has increased our ability to taxonomically identify specimens, examine genetic relatedness to contemporary varieties, infer various functional and phenotypic characteristics of ancient specimens and study the history of plant domestication^2^. Nevertheless, finding suitable sources of ancient DNA (aDNA) in plant species has been problematic due to the rapid decomposition of most plant material and the presence of PCR inhibitors in preserved material such as wood and seeds^3^,^4^.

The most abundant sources of plant archaeological material available are charred^5^,^6^,^7^,^8^. Thousands of charred seeds have been found in numerous archaeological sites under different states of preservation, with some deposits as old as the Stone Age^9^. The utility of charred material in archaeogenetics is questionable, with studies reporting variable success^1^. Experimental studies on modern material have shown the extent of damage in charred material is due to a combination of temperature, time and oxidation^10^,^11^,^12^. Ancient remains have the added disadvantage of DNA degradation accumulating over time^13^. Despite such damage, several studies have reported successful extraction and amplification of DNA from charred plant material from a range of species including peas^3^,^14^, wheat^6^,^15^,^16^, rice^17^, grapes^18^, maize^19^ and radish^20^. Yet other studies reported failure to amplify authentic DNA from charred material^21^,^22^,^23^,^24^, suggesting a high degree of stochasticity in successful experiments, compounded by the likelihood of bias toward publishing positive results^25^ and the absence of a formal method to assess the extent of charring.

A major hurdle when working with charred plant DNA, both modern and ancient, has been that the short DNA fragment lengths, typically <60 bp, are difficult to amplify via PCR^2^,^26^. High-throughput sequencing (HTS) overcomes this limitation, allowing for sequencing of very short DNA fragments^27^. Moreover, techniques such as target enrichment now allow for the preferential sequencing of DNA sequences of interest, regardless of fragment length^28^,^29^,^30^,^31^,^32^. Owing to these benefits, a combination of these techniques has been suggested as the future method of choice when working with charred, archaeobotanical material^2^,^8^. To date, the only study to examine the use of HTS on charred material describes successful recovery of barley, wheat and millet sequences from a 3300-year-old charred cereal assemblage, and discusses the potential for techniques such as target enrichment to enable sequencing of specific genes or regions of interest^8^.

Here we have combined the results of independent studies of four domesticated plant species *Hordeum vulgare* L (barley), *Vitis Vinifera* L (grape)*, Zea mays* L (maize) and *Oryza sativa* L (rice) using a combination of shotgun sequencing (all species) and target enrichment (barley, maize and rice) in order to assess the utility of HTS in aDNA studies of charred plant material. The specimens used range in age from 4450 calibrated years before present (YBP) to 550 YBP and represent a range of preservation states commonly encountered at archaeological sites. Our aim was to determine whether we could generate sufficient, authentic data from charred material using HTS that allows further downstream analyses relevant to the fields of archaeology and biology. We further re-analysed a study that has reported endogenous DNA from charred cereal grains over 3000 years old^8^.

## Results

### Read characteristics

The number of raw DNA sequencing reads (Illumina technologies) obtained ranged from 135 982 in Maize3 to 43 339 302 in Blank_maize1 for shotgun libraries (Table 1). Read lengths were constrained by the sequencing mode, although were generally short, with averages ranging from 63 bp to 228 bp (Table 1). The percentage of mapped reads identified as duplicates was highly variable across both species and samples, ranging from 0% (Barley3a, Barley8, Rice9) to 94.5% (Rice15) (Table S1). On average, duplicates were nearly five times more prevalent in enrichment libraries (average of 40.8% of reads mapped) than in samples that were shotgun sequenced (average of 7.9%).

### Reads mapped to the reference genomes

Post-filtering, the percentage of reads that mapped to sample genomes ranged from 0% (Rice 10 and Rice12a) to 0.12% (Barley5) (Table 1). There was no significant difference between the number of reads mapped to the respective genomes in libraries that had been enriched (independent of enrichment method) as opposed to shotgun sequenced when standardised for sequencing effort (Mann-Whitney *U* = 193, n1 = 24 n2 = 20, P > 0.05 two tailed). Although statistical testing was not possible due to small sample sizes, when comparing the different enrichment methods applied to the rice libraries, we observed more reads mapping in the target enriched libraries (avg. 0.01%), followed by whole genome (WG) enriched libraries (avg. 0.004%) and solid-state (SS) enriched libraries (avg. 0.0008%) (Table 1).

When mapping reads to the genomes of the other taxa included in the study, a higher percentage of reads were found to map to other, non-target genomes compared to the percentage of reads mapping to the target genome in the majority of cases (Table 2). In particular, on average, a greater proportion of reads mapped to the barley genome with 35 of our 51 samples mapping better to barley than to the other genomes (Table S2). The least amount of reads mapped to the rice genome (Table 2).

### aDNA damage patterns and sample bleeding

The majority of samples did not yield reads with the typical fragmentation and mis-incorporation patterns associated with aDNA, though for samples with few aligned reads there was insufficient data to obtain meaningful distributions from mapDamage. Nonetheless, for those with sufficient data, a total of 15 libraries yielded reads that showed typical aDNA damage patterns after aligning these reads to the grape genome (Supplementary Information, Table S2 and Fig. S2). These reads were sequenced from libraries of three grape samples, seven maize samples (including both libraries from Maize8), one maize extraction blank and three barley extraction blanks. *Irrespective of the sample origin*, aDNA damage patterns were *only* observed when reads were mapped to the grape genome, and not when mapped to any of the other three genomes included in this study. Furthermore, all libraries in which we observed these aDNA damage patterns were sequenced at the Danish National High-throughput DNA Sequencing Centre, and were sequenced in pools that contained additional libraries from non-charred ancient grape samples with high (10% to 69%) endogenous DNA that were not part of the present study (as well as libraries from non-charred maize with low endogenous DNA and libraries from other taxa). Given that aDNA damage patterns were only observed in those samples that were sequenced together with ancient grape samples (with high endogenous DNA content), we investigated if these damage patterns could originate from reads that were falsely assigned to the charred samples, i.e. due to “sample bleeding”. Sample bleeding is a recognized, but arguably underappreciated technical error caused by Illumina hardware and software, leading to a very small proportion of reads erroneously being assigned to another sample in a multiplexed run^33^ (see discussion and methods). By directly observing the index of short mapped reads (Supplementary Information Fig. S3)^33^, we indeed found a significant increase of incorrect-index assignments of non-charred grape samples in those reads that mapped to the grape genome in the 15 libraries with aDNA damage patterns. We could trace 4% to 39% of these reads back to non-charred grape samples with high endogenous DNA sequenced in the same pool. Such levels of false-index assignment are orders of magnitude higher compared to the background levels of grape sample bleeding (between 0.002% to 0.09%) observed in the non-mapping read data (Wilcoxon Signed Rank, *W* = 0, *N* = 16, *p* < 0.05) (Table S3). In other words, the grape aligned read data in these charred samples and these extraction blanks are significantly more likely to have originated from erroneous aDNA sources compared to other reads in these libraries.

### Metagenomic analysis

The majority of reads generated from the four species libraries were either bacterial in origin (43–72%) or unassignable (21–55%) according to analysis with BLASTn and MEGAN (Table 3). This was followed by hits to eukaryote genomes (2–8%), other plants (0.4–2%) and the target species (0.01–0.16%). An average of 84.7% of reads generated from the extraction blanks were unassignable, with the rest determined as mostly bacterial in origin. Detailed assessments of each sample are presented in the supplementary information (Supplementary Information Table S4).

### PIA filtering

All metagenomic BLASTn analyses produced hits on the target species, but following Phylogenetic Intersection Analysis (PIA^34^) and filtering for low taxonomic diversity within the larger landscape of BLAST hits plus further filtering for coverage length (95% and 99%), only eight samples retained positive hits. These samples were Barley1a, Barley5, Grape3, Grape4, Grape5, Maize5 (all 1–3 hits) and Maize8a (54 and 37 hits for 95% and 99% coverage respectively) and Maize8b (114 and 82 hits for 95% and 99% coverage respectively; Table 4). The Maize8 library that had been subjected to target enrichment (Maize8b) produced twice as many hits as the library that was shotgun sequenced with similar sequencing depth (Table 4). Of the PIA filtered reads from Maize 8a (shotgun library), 11 of the 54 (95% coverage) and 1 of the 37 (99% coverage) were non-duplicate reads that mapped back to the maize genome. For Maize 8b (capture library), 15 of the 114 (95% coverage) and 1 of the 82 (99% coverage) were non-duplicate reads that mapped back to the maize genome.

BLASTn analyses of the 496 purportedly endogenous reads from Bunning *et al*.^8^ resulted in a majority of reads producing hits to *Mus musculus* (domestic mouse; Supplementary Information Table S5). Only 0.2% were assigned to one of the listed taxa, *Hordeum vulgare*, but none of these reads remained following PIA filtering. Using RepeatMasker we further identified 195 of the 496 sequences as containing regions of low complexity or simple repeats (Supplementary Information Table S6).

## Discussion

PCR-based aDNA studies have highlighted the difficulties of working with charred archaeobotanical remains, showing that endogenous DNA is often inaccessible, and highly damaged when it is recovered^8^. While HTS has opened new doors to paleogenomic approaches for many species and tissue types, HTS of charred archaeobotanical specimens remains relatively unexplored. We have evaluated shotgun HTS and target enrichment in independent studies of 38 archaeological remains from four species, and failed to retrieve sufficient authentic DNA data to address basic archaeological and biological questions from our specimens. Given that most ancient plant remains are preserved via charring, this is an especially disappointing revelation. Below we discuss the problems associated with the low or absent endogenous DNA content present in charred specimens and argue the need for thorough analytical approaches to avoid spurious conclusions on DNA authenticity.

Using laboratory and analytical pipelines optimized for aDNA, we found exceptionally low percentages of reads (from 0 to 0.12%) mapping to the target genomes in all 38 samples. Low endogenous DNA content is a common characteristic of ancient DNA specimens^27^ with values often falling below 1%^35^. This in itself does not preclude the presence of authentic reads in samples, although it does require an often prohibitory amount of sequencing in order to yield sufficient data for meaningful downstream analyses^35^.

In order to evaluate the authenticity of our data we mapped all sample reads to the genomes of the three other taxa used in this study. Short reads that may contain sequence errors or mutations are notoriously difficult to align and can result in their mapping to multiple locations within a genome or even to multiple genomes^36^,^37^. We observed similar, and at times greater numbers of reads mapping to the non-target genomes. Moreover, in every case the extraction blanks, on average, had a higher percentage of reads mapping to all four genomes than the respective specimen samples. Preferential mapping of short reads to certain genomes may depend on factors such as genome size and complexity. In our study, the average highest percentage of reads (regardless of sample origin) was found to map to the large barley genome (5.3 Gbp), whereas the lowest percentage mapped to the smaller rice genome (0.4 Gbp). The mapping of short reads to multiple genomes does not necessarily preclude the presence of authentic DNA^36^, yet when reads map equally well or better to a number of unrelated genomes, the authenticity of the reads is questionable. The reads mapping to the target genomes may therefore not represent endogenous DNA and instead may be an artefact of spurious mapping of short reads.

Analysis of DNA damage can also be used to support the authenticity of the reads obtained. In our case the use of mapDamage served to highlight another issue that can arise when working with very small numbers of reads–that of the potential for sample bleeding to occur between samples run on the same sequencing lane^33^. Sample bleeding can occur via two processes, the introduction of errors during PCR or sequencing, or over-clustering/mixed clusters on the flow cell^33^,^38^. The former issue can be mitigated by using indexes that are dissimilar, for example differing by at least 3 nucleotides, a strategy employed in this study. The latter issue is caused by incorrect index assignment on the Illumina flowcell, leading one read cluster from a sample being assigned the index of a neighbouring read cluster from another sample. This problem is not detectable in most cases, and should have negligible impact when high coverage filters are implemented in downstream analyses. The identification of what appeared to be authentic ancient grape reads based on damage patterns in 15 of our samples (three charred grape, seven charred maize and four extraction blanks), was found to be associated with significant increases in false-index assignments, linking these damage patterns to reads from uncharred ancient grape samples with high endogenous DNA content sequenced in the same pool, rather than to the samples themselves. False-index assignments (sample-bleeding) occurs in an estimated ~0.3% of reads when using single-indexed libraries^38^, and in most circumstances sample-specific, endogenous DNA reads greatly outnumber such erroneously assigned reads. However, false-index pairings can be particularly problematic in studies that assess rare variants or when emphasis is placed on a highly limited number of reads^38^. In our case, we observed less than 0.3% of reads aligning to any of the respective reference genomes, which makes our data particularly vulnerable to the confounding effects of false-index assignments. We caution researchers in the aDNA field against making conclusions based on very low read numbers particularly when samples have been pooled together with high endogenous ancient libraries of the same taxa.

Analysis of the metagenome has begun to receive more attention in recent aDNA studies allowing for additional information to be obtained from samples^39^,^40^,^41^,^42^. When HTS output of libraries from charred material indicates low endogenous content, metagenomic analysis may, for example, reveal misidentification of the archaeological specimen. Charring can impact seed morphology, hampering specimen identification, particularly when working with mixed charred assemblages^1^,^43^. In these cases, if endogenous DNA remains, the metagenome may reveal the true specimen identity. Metagenomic analysis of our samples indicated the majority of reads were microbial in origin or un-assignable, with the remainder identified as either eukaryotic or plant in origin. This contrasts to another published metagenome analysis of charred grains which identified the majority of reads as metazoan in origin followed by green plants^8^ (but see below). Very few reads produced BLAST hits to the target species in our study (less than 0.2%) and these were filtered using PIA to validate authenticity. PIA filtering is robust at identifying or dismissing sequence data as probabilistically ‘genuine’ from BLAST outputs, due to its consideration for database bias in favour of model organisms and its ability to identify threshold-scoring yet spuriously-assigned reads with only superficial similarity to their closest database match (see ref. 34 for further details). The PIA algorithm also gives a probabilistic assignment to a given sequence recursively at descending taxonomic ranks (i.e. where a read matching barley can be dismissed as being conclusively *Hordeum* sp., it might be confidently assigned at a higher taxonomic rank). PIA filtering indicated that in our data only eight of the 44 samples contained sequences that passed justifiably stringent levels of filtering. Of these, six of the samples (two barley, three grape and one maize) were left with so few remaining reads (≤3) that we would consider them inconsequential, particularly given the possibility of contamination (see below). Two of the maize libraries – represented by one shotgun and one target enrichment library of the same specimen - retained higher numbers of reads post-filtering with 95% coverage, which although less stringent than the 99% filter may better account for near-terminal cytosine deamination. However, further investigation revealed that of the reads that could be mapped back to the maize genome, many were duplicates leaving just 11 non-duplicate reads from the shotgun library and 15 non-duplicate reads from the capture library (26 reads total from Maize8 specimen). The presence of duplicates in the filtered data was surprising given these samples had previously been run through duplicate removal software and highlights the importance of cross checking all data post-analysis. The 26 non-duplicate reads were derived from a portion of a cob dated to circa 3960 YBP. This specimen had been identified as lightly charred and it could be that less charred portions of the cob retained endogenous DNA, warranting further investigation (Fig. S1).

By mapping our data to other genomes, using PIA filtering of our BLASTn results, and further analysing these results, we have rigorously tested the authenticity of all putatively endogenous reads in our dataset, showing extremely low success rates in our samples. Bunning *et al*.^8^ reported 496 reads from barley, einkorn, emmer and broomcorn millet from a 3300-year-old charred grain assemblage using SOLiD 5500 sequencing. Our reanalysis of these reads using the current NCBI database does not support this conclusion, with the majority of reads receiving either no hits or producing hits to *Mus musculus*. Although 0.2% of reads produced hits to barley, none of these remained following PIA filtering. Further analysis showed over 40% of the reads were identified as either low complexity or as containing simple repeats, which can produce spurious results in BLAST database searches^44^. Our reanalysis of these data differed from the published work in that we BLASTed against the entire NCBI database as opposed to using a specific cereals database. This discrepancy emphasizes the great impact of reference database selection in aDNA analyses and the corresponding repercussions on reliable taxonomic assessments.

Metagenomic profiles of extraction blanks provide possibilities to identify the level and nature of contamination present in lab reagents used^45^. Metagenomic analysis of the extraction blanks used in our study indicated fewer microbial reads than the charred sample libraries. The extraction blanks were in fact relatively low in bacterial DNA in comparison to control samples sequenced from other laboratories, as assessed in Salter *et al*.^45^. That study showed the composition of common laboratory contaminants to consist of over 90% bacterial reads^45^. The relatively low bacterial presence in our extraction blanks may reflect the effect of the stringent precautions taken in aDNA laboratories compared to laboratories where contemporary samples are analysed. Alternatively the unassigned hits may also be bacterial in origin but are underrepresented in the sequence database.

Contamination of ancient samples with modern DNA remains a serious concern, necessitating adherence to strict precautions when working with aDNA^46^. Despite the most stringent efforts, however, contamination can still occur and may be more likely when working with ubiquitous commercial crops, such as members of the *Triticeae*^47^,^48^. This is particularly problematic given many charred archaeobotanical studies aim to investigate aspects of historical domestication and the spread of agriculture in these species^26^. Even if the aim of analysing charred material is to taxonomically identify the charred specimen, the risks of underlying contamination and hence false positives needs to be considered. To better understand this issue, future studies that examine the background levels of contamination by modern crops in deep-sequenced datasets would provide a useful baseline for understanding and potentially quantifying this risk.

Although charring and carbonization is known to destroy DNA, the process by which this occurs is less well understood^1^,^7^,^10^. Fully carbonized material, where remains have been completely converted to inorganic material, is expected to be devoid of endogenous DNA^2^. Assessing the degree of charring in archaeological specimens prior to processing is difficult, but recent insights into the changing carbon and nitrogen isotope values over different charring conditions may help archaeologists assess the degree of charring prior to use in aDNA studies^49^. Larger plant structures may not always be charred throughout, allowing for the persistence of small amounts of endogenous DNA. Accordingly, the one sample in our study from which we may have retrieved authentic target DNA was a maize cob, which is larger than rice, barley or grape seeds. Nevertheless, the number of reads retrieved from this sample is extremely low and too little for any further analyses on functional traits or demography, which would require several orders of magnitude more data.

Although we used the most optimal protocol currently known for extracting DNA from botanical remains^50^ for three of the taxa (barley, maize and grape), advances in aDNA extraction methodology are continually improving the volume and size distribution of DNA data obtained from archaeological material^51^. Future studies of charred material may benefit from testing protocols specifically aimed at recovering ultra short DNA fragments (<50 bp, e.g. ref. 52).

## Conclusion

Charring is the most common form of archaeobotanical preservation, yet such remains have long vexed aDNA researchers due to inconsistent success, beyond what is commonly observed in most aDNA studies^1^. HTS and target enrichment have been suggested as promising solutions that would enable the recovery of aDNA from charred remains, ultimately providing the technology to investigate a range of archaeological and biological questions. Regrettably, we report that based on our independent studies of four plant species and the reanalysis of an earlier dataset, charred plant material appears to be largely incompatible with these technologies. For a combined cost of over 16 000 € (laboratory and sequencing costs only), our four studies have yielded a total of 26 potentially authentic sequences from one lightly charred specimen, out of a total of more than 200 million reads and 38 unique samples. Coupled with the substantial investment in time and money required to process charred samples we expect most HTS experiments of charred material will not yield sufficient reliable genetic data. We urge a great degree of caution to future researchers who would invest in charred material for archaeogenetic purposes and suggest all data be carefully scrutinized for false-positives resulting from non-stringent analyses or originating from contamination. Future studies that develop a cost effective means of evaluating the degree of charring present in archaobotanicals prior to their processing may provide useful developments in the field.

## Materials and Methods

### Samples

#### Barley

**Figure 1:**
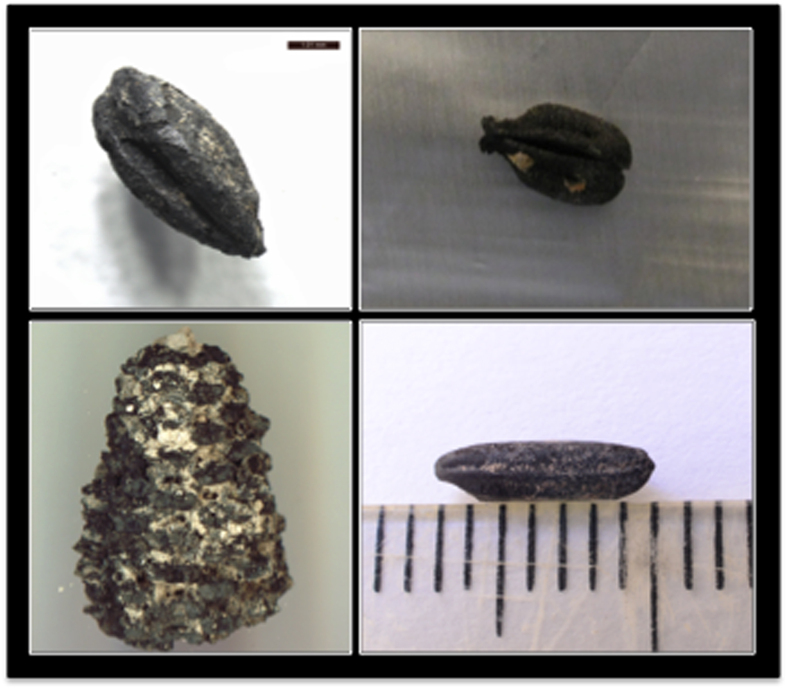
Examples of the charred material for each of the four species used in this study. Clockwise from top left: barley seed (*Hordeum vulgarum*), grape seed (*Vitis vinifera*), maize partial cob (*Zea mays*) and rice grain (*Oryza sativa*) (scale bar indicates mm).

Eight archaeological barley seeds, four excavated from Quoygrew, Orkney islands (950–850 calibrated YBP, from a well-stratified midden deposit^53^) and four from Kaupang, southern Norway (ca. 1150 YBP from waterlogged pitfalls^54^) were provided by the University of Cambridge and the Museum of Cultural History, University of Oslo, respectively. Seeds were light, fragile and appeared to be partially or fully carbonised based on colour and composition (Fig. 1).

#### Grape

Five archaeological grape seeds originated from Tell Tayinat, in southern Turkey. Two of the seeds were fully carbonised and dated based on stratigraphy and association with diagnostic artefacts to the Early Bronze Age, ca. 4450–3950 calendar YBP, and the remaining three seeds were less carbonised and dated to the Iron Age ca. 3050–2500 YBP (pers. com. Doga Karakaya) (Fig. 1).

#### Maize

A total of eight archaeological maize samples were tested within this project. One sample was excavated from the Montoya Site in the Cañada Alamosa, New Mexico and provided by Human Systems Research. A portion of the partially charred cob has been directly AMS dated to 3925 calibrated YBP. The seven other maize samples were excavated and provided by Arizona State University. Three heavily carbonised specimens consisting of cobs with attached kernels come from Barton Creek Cave, a Maya site from the Late to Terminal Classic Era (ca. 1350–950 YBP) in the Cayo District of Western Belize. Three cobs come from Non-Grid 4, an Epiclassic human sacrifice shrine site in the Northern Basin of Mexico (ca. 1350–1050 YBP). An additional heavily charred cob originates from a chinampa canal at Xaltocan, a Postclassic site (ca. 750–550 YBP) in the Northern Basin of Mexico (Fig. 1).

#### Rice

Grains from a total of seven archaeological accessions from sites across the Indian subcontinent, Thailand, and the Comoros Islands were excavated and provided by the University College London and Oxford University. The carbon-14 calibrated dated sites included Sima (Comoros; ca. 1265–965 YBP), Ter, Balathal (India; ca. 2145–1990 YBP and 2345–2155 YBP), Ban Non Wat (two contexts; ca. 2655–2185 YBP), Noen Ul Loek (ca. 1695–1535 YBP and Non Ban Jak (Thailand; Iron Age). All samples were light, porous, fragile and heavily carbonised (Fig. 1).

### DNA extractions

All DNA extractions and library builds were carried out in dedicated ancient DNA laboratories at the University of Oslo (barley), the University of Copenhagen (grape and maize) and University of Warwick (rice and three barley seeds); all of which adhere to the highest standards of aDNA quality control^46^. Originally, these were four independent experiments not intended for publication together and as a result methods vary amongst species and laboratories.

The barley, grape and maize samples were extracted using the methodology of Wales *et al*.^50^,^55^. Treatment of the charred material prior to extraction and minor modifications made are detailed in the Supplementary Information. The rice was extracted using a modified DNEasy protocol (Qiagen) (see Supplementary Information for details). All extraction experiments included negative controls.

### Library Preparation

DNA libraries of barley extracts were built using both a single stranded (ss) DNA library preparation protocol^56^ and a double stranded (ds) DNA library preparation protocol^57^. For both library builds, sample-specific seven bp indexes in the P7 primer were used^57^. Details on the library preparation are provided in the Supplementary Information.

DNA libraries for grape and maize were also constructed using dsDNA library preparation protocol^57^. The method was similar to that used for the barley samples, but with a few differences in reaction volumes and purification strategies described in the Supplementary Information.

Rice libraries were constructed using Illumina TruSeq Nano kits, following the manufacturer’s instructions. Modifications are listed in the Supplementary Information.

### Target Enrichment

Six barley libraries (four built using the ss protocol and two using the ds protocol) were subjected to target enrichment using a custom-designed MYbaits kit (MYcroarray, Ann Arbor, Michigan) consisting of 25029 biotinylated RNA probes (80-mer length, 4 x flexible tiling density). For rice, three separate enrichment approaches were applied: whole-genome in-solution, targeted in-solution, and solid-state targeted that utilises an array chip for hybridization (Table 1). All formats were supplied by MYcroarray. One maize specimen (Montoya) was enriched for 348 genes using a targeted in-solution hybridization MYbaits kit (MYcroarrary). Details of the target enrichment design can be found in the Supplementary Information.

### Sequencing

HTS platforms used for each library are provided in Table 1. Sequencing of the libraries was carried out at the Norwegian Sequencing Centre (barley), Danish National High-throughput DNA Sequencing Centre (grape, maize and barley extraction blanks) and the University of Warwick (rice). See Supplementary Information for more details on quantification and pooling.

### Data Filtering and Analysis

Raw reads from all four species were collapsed (when paired-end sequenced), trimmed of adapters and truncated where necessary using AdapterRemoval v. 2.1.2^58^ with the following settings: –qualitybase 33 –minlength 25 –mm 3 –trimns –trimqualities. Reads from each sample were mapped against the following four reference genomes: *Hordeum vulgare* 082214v1.29, *Zea mays* AGPv3.30, *Vitis Vinifera* IGGP_12 × 30, *Oryza sativa* IRGSP-1.0.30 downloaded from the ENSEMBL database. To evaluate the authenticity of the mapped reads, all reads were mapped to all four genomes used in the study. Reads were mapped using the bwa *aln* and *samse* algorithms, with seeding disabled and -o 1 and -n 0.03, following recommendations in Schubert *et al*.^59^. SAM files were converted to BAM files and sorted using Samtools v1.1, keeping only those reads with a minimum mapping quality score (MapQ) of 25. Duplicates were removed with MarkDuplicates from Picard Tools v.1.96 (http://picard.sourceforge.net/). Finally, we obtained aDNA damage patterns using mapDamage v.2.0.6^60^,^61^ for reads from all libraries mapped to each of the four genomes (i.e. from four BAM files per library). After observing aDNA damage patterns in several libraries when mapped to the grape genome *only* (see results) we investigated the potential for false-index assignment (i.e. sample bleeding)^33^ from non-charred grape samples that were sequenced in the same pool. The Illumina platform uses a separate set of index cycles to read the sample-specific index, and computationally assigns sequencing reads to their respective sample based on that data. Nonetheless, the sequencing cycles may directly observe the index in those cases where the insert is short, leaving sufficient cycles to pass the Illumina specific P7 adapter and the index itself (our data required 38 cycles to cross the P7 adapter and the index, see also Supplementary Information and Fig. S3). It was therefore possible to compare the index used during demultiplexing (i.e. read by the index cycles) to the one in the actual sequencing data (read by the sequencing cycles when inserts are sufficiently short). Hence, we observed the indexes generated by the sequencing cycles in all libraries showing aDNA damage when mapped to grape. In these libraries, we calculated the fraction of correct indexes and the fraction of indexes that belonged to the high endogenous, non-charred grape samples that had been sequenced in the same pool. We did this analysis for both the unaligned sequencing data and the grape aligned BAM files. For this analysis, we only used reads that were short enough for the P7 adapter and the index to be fully sequenced (i.e. read-length minus 40 bp), and we used simple, exact pattern matching (unix; grep) to identify indexes in these sequencing reads. Because HiSeq sequencing data typically has increased levels of sequencing error at read-ends^62^, requesting an exact match of the entire adapter sequence including the index would fail to identify many instances of the adapter and the index. We therefore counted all reads that contained an exact match of the first 12 bp of the P7 adapter (confirming the presence of the adapter in the sequence reads), followed by an exact 6 bp match of the specific index under investigation. This approach allowed for sequence variation in the adapter sequence between the first 12 bp of the adapter and the 6 bp index (a 22 bp stretch).

### MEGAN and PIA

Exact duplicates were removed from raw fastq data using Prinseq Lite v. 0.20.4 (-derep 1 -derep_min 2)^63^. Files were subsequently converted into fasta format and subjected to metagenomic BLAST using the complete NCBI nucleotide database (downloaded 19/02/2015) on a standalone server. To avoid over-sensitivity or over-stringency, default values for seed size and the scoring matrix were used for the BLASTn algorithm. BLAST output was tabulated with taxon IDs appended for downstream analysis. Complete BLAST outputs for each sample were imported into MEGAN 5^64^ using the default parameters. For each sample, reads from the terminal node were exported for the species in question (i.e. barley, grape, maize or rice). Sequence data for each read ID was then recovered from the original data files using the Unix grep function and run through the BLAST program again, using default output format to obtain read length data for downstream analysis. These reads were further analysed using phylogenetic intersection analysis (PIA^34^) to obtain taxon diversity information and further filtered according to read length coverage^34^ disregarding reads with less than 95% or 99% coverage to their closest database match. We included results from both 95% and 99% coverage filtering as although the 99% filter is more stringent, the 95% filter may better account for near-terminal cytosine deamination, typical of aDNA. After filtering with PIA we double-checked the quality of the remaining reads in Maize8a and Maize 8b by mapping these back to the maize genome and removing duplicates using the methodology described above.

In addition, we accessed the 496 reads of Bunning *et al*.^8^ that were reported as reads from barley, einkorn, emmer and broomcorn millet and we reanalysed these data using BLASTn and PIA. The sequences were also run through RepeatMasker^65^ using default settings to assess whether reads could be classified as containing either simple repeats or low complexity.

## Additional Information

Accession codes: All individual read data are available at the European Nucleotide Archive (ENA, www.ebi.ac.uk/ena) under study accession number PRJEB15180. 

**How to cite this article**: Nistelberger, H. M. *et al*. The efficacy of high-throughput sequencing and target enrichment on charred archaeobotanical remains. *Sci. Rep.* **6**, 37347; doi: 10.1038/srep37347 (2016).

**Publisher's note:** Springer Nature remains neutral with regard to jurisdictional claims in published maps and institutional affiliations.

## Supplementary Material

Supplementary Information

## Figures and Tables

**Table 1:** Sample details and characteristics including: sequencing platform, paired-end (pe) or single-end (se), maximum read length (bp); whether libraries were shotgun sequenced or subjected to target enrichment, whole genome (WG) or solid-state(SS); double stranded (ds) or single stranded (ss) library build; average read length (bp); total number of raw reads, or read pairs if paired-end; percentage of reads that mapped to sample genomes after duplicate removal.

Sample	HTS platform	Method	Library build	Avg. read length (bp)	# Raw reads	% Reads mapped to target
Barley1a	HiSeq2500/pe/125 bp	target enrichment	ds	92	9363464	0.008
Barley1b	Miseq/pe/75 bp	target enrichment	ss	78	4052951	0.029
Barley2a	HiSeq2500/pe/125 bp	target enrichment	ds	100	6943249	0.007
Barley2b	Miseq/pe/75 bp	target enrichment	ss	76	4577402	0.040
Barley3a	HiSeq2500/pe/125 bp	shotgun	ds	80	1445024	0.010
Barley3b	Miseq/pe/75 bp	shotgun	ss	72	5006428	0.022
Barley4a	HiSeq2500/pe/125 bp	shotgun	ds	95	6406123	0.008
Barley4b	Miseq/pe/75 bp	shotgun	ss	83	353474	0.014
Barley5	Miseq/pe/75 bp	target enrichment	ss	69	3641058	0.123
Barley6	Miseq/pe/75 bp	target enrichment	ss	68	3031164	0.085
Barley7	Miseq/pe/75 bp	shotgun	ss	68	2384691	0.076
Barley8	Miseq/pe/75 bp	shotgun	ss	78	639883	0.015
Grape1	HiSeq2500/se/100 bp	shotgun	ds	69	6337515	0.003
Grape2	HiSeq2500/se/100 bp	shotgun	ds	72	8657842	0.003
Grape3	HiSeq2500/se/81 bp	shotgun	ds	72	6491713	0.008
Grape4	HiSeq2500/se/81 bp	shotgun	ds	67	9147375	0.009
Grape5	HiSeq2500/se/81 bp	shotgun	ds	66	5794427	0.015
Maize1	HiSeq2500/se/81 bp	shotgun	ds	72	5052523	0.006
Maize2	HiSeq2500/se/81 bp	shotgun	ds	73	7351039	0.007
Maize3	HiSeq2500/se/81 bp	shotgun	ds	67	135982	0.011
Maize4	HiSeq2500/se/81 bp	shotgun	ds	65	10742918	0.013
Maize5	HiSeq2500/se/81 bp	shotgun	ds	66	7822641	0.016
Maize6	HiSeq2500/se/81 bp	shotgun	ds	67	7645073	0.012
Maize7	HiSeq2500/se/81 bp	shotgun	ds	67	6588747	0.007
Maize8a	HiSeq2500/se/100 bp	shotgun	ds	72	28239900	0.026
Maize8b	HiSeq2500/se/100 bp	target enrichment	ds	78	21895732	0.016
Rice1	MiSeq/se/250 bp	target enrichment WG	ds	193	390515	0.005
Rice2	MiSeq/se/250 bp	target enrichment	ds	63	500538	0.015
Rice3	MiSeq/se/250 bp	target enrichment SS	ds	141	1783367	0.001
Rice4	MiSeq/se/250 bp	target enrichment WG	ds	228	499372	0.001
Rice5	MiSeq/se/250 bp	target enrichment SS	ds	114	4953182	0.001
Rice6	MiSeq/se/250 bp	target enrichment WG	ds	219	567506	0.002
Rice7	MiSeq/se/250 bp	target enrichment SS	ds	131	6101994	0.001
Rice8	MiSeq/se/250 bp	target enrichment WG	ds	194	447690	0.002
Rice9	MiSeq/se/250 bp	target enrichment WG	ds	227	301980	0.002
Rice10	MiSeq/se/250 bp	target enrichment SS	ds	177	3425508	0.000
Rice11	MiSeq/se/250 bp	target enrichment WG	ds	172	592182	0.013
Rice12a	MiSeq/se/250 bp	shotgun	ds	206	2608916	0.000
Rice12b	MiSeq/se/250 bp	target enrichment WG	ds	179	1476971	0.001
Rice13	MiSeq/se/250 bp	target enrichment	ds	70	396830	0.012
Rice14	MiSeq/se/250 bp	target enrichment	ds	74	1096766	0.018
Rice15	MiSeq/se/250 bp	target enrichment	ds	78	595556	0.006
Rice16	MiSeq/se/250 bp	target enrichment	ds	64	338135	0.009
Rice17	MiSeq/se/250 bp	target enrichment	ds	93	373420	0.002
Blank_barley1	HiSeq2500/se/81 bp	shotgun	ds	54	33740465	NA
Blank_barley2	HiSeq2500/se/81 bp	shotgun	ds	66	7126362	NA
Blank_barley3	HiSeq2500/se/81 bp	shotgun	ds	68	671521	NA
Blank_grape1	HiSeq2500/se/100 bp	shotgun	ds	58	33090380	NA
Blank_maize1	HiSeq2500/se/100 bp	shotgun	ds	52	43339302	NA
Blank_maize2	HiSeq2500/se/81 bp	shotgun	ds	71	15727571	NA
Blank_rice1	MiSeq/se/250 bp	shotgun	ds	91	250044	NA

**Table 2:** Average percentage of reads from all barley, grape, maize, rice and blank samples that map to the four reference genomes.

Samples	Reference genome			
	Barley	Grape	Maize	Rice
	5300 Mbp	500 Mbp	2500 Mbp	430 Mbp
Barley samples	0.036	0.019	0.025	0.022
Grape samples	0.018	0.007	0.021	0.012
Maize samples	0.018	0.011	0.013	0.009
Rice samples	0.010	0.008	0.004	0.005
Extraction blanks	0.051	0.037	0.038	0.028

Genome size is listed under each reference genome in megabase pair (Mbp).

**Table 3:** Percentage taxonomic assignments of reads averaged across each species and the series of blanks determined using MEGAN.

	Barley	Maize	Grape	Rice	Blanks
Bacteria	55.66	67.25	72.26	42.92	9.36
Eukaryotes	4.06	8.06	4.59	1.82	5.74
Plants	0.65	1.62	2.04	0.36	0.19
Target	0.01	0.14	0.16	0.03	0.00
Not assigned	39.61	22.94	20.95	54.86	84.70

Results indicate assignment prior to PIA filtering.

**Table 4:** Results of BLASTn and Phylogenetic Intersect Analysis (PIA) showing the number of reads BLASTED after duplicate removal using Prinseq, the number of metagenomic BLAST hits on the sample species, Post PIA filtering hits at 95% coverage (>0.2 taxon diversity) and 99% coverage (>0.2 taxon diversity).

Sample	# Reads	BLAST hits target species	Post-PIA hits (95%) cvg.	Post-PIA hits (99%) cvg.
Barley1a	8433351	73	2	2
Barley1b	2855828	20	0	0
Barley2a	6525081	64	0	0
Barley2b	3163453	18	0	0
Barley3a	1415373	13	0	0
Barley3b	4001313	9	0	0
Barley4a	6019807	40	0	0
Barley4b	242478	1	0	0
Barley5	2561257	22	1	1
Barley6	2283844	4	0	0
Barley7	1741206	7	0	0
Barley8	409890	3	0	0
Grape1	6157219	32	0	0
Grape2	8477672	28	0	0
Grape3	6315327	755	2	1
Grape4	5685642	873	3	3
Grape5	8672311	744	1	0
Maize1	4911944	2	0	0
Maize2	7254256	1	0	0
Maize3	88796	0	0	0
Maize4	8530317	71	0	0
Maize5	5396523	128	1	1
Maize6	6446171	74	0	0
Maize7	6479527	2	0	0
Maize8a	27518813	2714	54	37
Maize8b	14715580	3096	114	82
Rice1	390515	0	0	0
Rice2	500538	0	0	0
Rice3	1783367	13	0	0
Rice4	499372	64	0	0
Rice5	4953182	0	0	0
Rice6	567506	0	0	0
Rice7	6101994	166	0	0
Rice8	447690	0	0	0
Rice9	301980	1	0	0
Rice10	3425508	99	0	0
Rice11	592182	26	0	0
Rice12a	2608916	150	0	0
Rice12b	1476971	66	0	0
Rice13	396830	217	0	0
Rice14	1096766	99	0	0
Rice15	595556	289	0	0
Rice16	338135	111	0	0
Rice17	373420	47	0	0
Blank_barley1	3754102	50	0	0
Blank_barley2	5390039	12	0	0
Blank_barley3	567952	24	0	0
Blank_grape1	2053566	13	0	0
Blank_maize1	2687850	371	0	0
Blank_maize2	10293162	11	0	0
Blank_rice1	250044	0	0	0
