# Supplementary material for: The efficacy of high-throughput sequencing and target enrichment on charred archaeobotanical remains
Source: Sci Rep. 2016 Nov 24;6:37347. doi: 10.1038/srep37347 (PMC5121605; doi:10.1038/srep37347)
Supplement: Supplementary Information [file srep37347-s1.doc]

**SUPPLEMENTARY INFORMATION**

**The efficacy of high-throughput sequencing and target enrichment on charred archaeobotanical remains**

H M Nistelberger, O Smith, N Wales, B Star and S Boessenkool

**Table Of Contents**

**Page**

Supplementary methods 2

DNA extraction 2

Library build 4

Target enrichment 6

Sequencing 7

Figure S1 Photograph of Montoya maize sample 9

Figure S2 Examples of aDNA damage patterns 10

Figure S3 Overview of sample bleeding analysis 11

Tables S1 Sample sequencing characteristics 12

Table S2 Number of reads mapping to each genome 14

Table S3 Proportion of bled indexes 15

Table S4 Assignment of reads using MEGAN 16

Table S5 Assignment of read data from Bunning et al 2012 17

Table S6 RepeatMasker read data from Bunning et al 2012 18

References 22

**Supplementary methods**

DNA extraction

Barley

Barley seeds were flushed gently with UV treated Millipore water to remove external residue, then crushed in individual 1.5ml UV-treated Eppendorf tubes using sterile, disposable minipestles. DNA was extracted from crushed seeds using a modified method of Wales et al., (2014, 2012)^1,2^ with the exception that samples were incubated overnight at 37**°**C rather than 55**°**C. A MinElute purification step was conducted after the phenol and chloroform extractions according to Dabney et al., (2013) ^3^. DNA was eluted in 20µl of elution buffer. Negative controls were included with every batch of seed extractions.

Grape

To reduce environmental contamination in archaeobotanical remains, samples are frequently washed for 30 seconds in a solution of 10% sodium hypochlorite (bleach), followed by a rinse in molecular biology-grade water to neutralize the bleach. Four seeds were processed in this manner, however, for one of these samples (Grape2) the seed sank into the solution, potentially absorbing bleach and degrading endogenous DNA. Therefore, one seed (Grape1) was only rinsed in water. Seeds were wrapped in UV-treated aluminium foil and crushed with a sterilized hammer. DNA was extracted following the protocol described in Wales et al., (2014, 2012) ^1,2^, using 1000 μL of digestion buffer. The MinElute purification step conducted after the phenol and chloroform extractions was performed following the method developed by Dabney et al., (2013) ^3^ to improve the recovery of short DNA fragments. A negative control was processed with the samples.

Maize

Due to the porous and fragile nature of the charred cobs and kernels, it was not possible to wash surfaces in bleach or water. Instead, maize specimens were cut with sterile scalpels to isolate portions of the cob that were least exposed to archaeological sediment. A mass of 41-274 mg of tissue was collected for each sample. Sampled tissues were placed in UV-treated 2.0 mL tubes and crushed with sterile minipestles. DNA was extracted in the same manner as grape samples, including an extraction blank.

Rice

DNA from rice was extracted with a modified DNeasy (Qiagen) protocol. The rice grains were too porous to wash with bleach or water, but also too small to cut useable sections from. Grains were crushed to powder using the blunt end of a sterile loop and immersed in CTAB buffer (2% CTAB, 1%PVP, 1.4M NaCl, 20mM EDTA) for 72 hours at 37°C. DNA was extracted once with an equal volume of 24:1 chloroform:isoamyl alcohol, mixed with 5x volume Buffer AW1, and incubated for two hours at room temperature. The solution was then passed through a DNeasy spin column, which was subsequently washed once with 500 μL buffer AW2, washed once with 300 μL acetone, and finally allowed to air dry to prevent loss of DNA by excessive forces resulting from centrifugation. DNA was eluted from the column in 50 μL elution buffer after a 15 min incubation.

Library Build

Barley

At total of 12 genomic libraries were built from the eight seeds; eight were prepared using a single-stranded library preparation method (see below), four of which were shotgun sequenced and four of which were subject to capture. Another four libraries were built using the double stranded library preparation method (see below), two of which were shotgun sequenced and two of which were subject to capture. For the single stranded (ss) method, deoxyuracils derived from cytosine deamination were not removed. For the double stranded (ds) method, we used the NEB next E6070Next® DNA library Prep Master Mix Set for 454 reagents as per ^4^, with the exception that all reactions were performed in half volumes. For both ss and ds methods positive and negative controls were included as per the original protocols. Library amplification was performed using *PfuTurbo* Cx Hotstart polymerase (Agilent; 12 cycles). Amplified library products were cleaned using Agencourt® AMPure XP beads at a 1:1.7 ratio (PCR volume: AMPure XP volume), eluted in 30µl sterile Millipore water and visualised and quantified on a Bioanalyzer 2100 (Agilent). Subsets of libraries were re-amplified to increase library concentration when required using Accuprime Pfx DNA polymerase (Invitrogen) and the primers IS5 and IS6 as per ^5^. Re-amplifications of libraries were split across four separate reactions to reduce PCR bias.

Grape and Maize

Libraries were built using 20 μL of DNA extract, except for one maize sample (Maize3). This DNA extract had unusually dark pigmentation which could likely lead to enzymatic inhibition during the library preparation, so only 10 μL of volume was used. Dilutions of the unamplified libraries were measured with a qPCR assay to determine how many PCR cycles were required to reach sufficient quantities of DNA for sequencing ^6^. Library amplification was performed with AmpliTaq Gold (ThermoFisher Scientific), using a sample-specific index in the P7 primer ^5^. PCR was done in duplicate for each sample, with each PCR using 20 μL of library in a 100-μL reaction. Amplified library products were purified with Qiagen QiaQuick spin columns, and evaluated on a TapeStation 2200 (Agilent).

Rice

Shotgun libraries for each sample were constructed with size selection to remove large (>300bp) fragments due to their likelihood of being environmental contaminants. Three iterations of DNA capture were employed; grains from all seven samples were used in the first capture iteration. Grains from all seven with the exception on Iron Age Ban Non Wat were used in the second, and Grains from Balathal, Iron Age Ban Non Wat and Non Ban Jak were used in the third; see ‘Target Enrichment’ for details of capture methodology. The following modifications were made for the Illumina TruSeq library builds. For very short insert sequences: 1) the end repair incubation step was extended to one hour to compensate for anticipated excessive damage due to the age of the samples, 2) cleanup steps were performed using Minelute (Qiagen) columns, 3) 5x binding buffer and acetone cleanup / drying was used as described for the rice DNA extractions above, 4) the adenylation and the adapter ligation incubation times were extended to 30 minutes. To prevent excessive adapter-dimer formation, we diluted adapters to 10% of manufacturer-recommended concentrations. SPRI-based size selection was performed after adapter ligation since fragments were now long enough for effective capture; we used a 0.3x bead ratio for removal of large contaminant fragments, and subsequent 1x ratio for isolation of desired fragments. PCR setup was performed according to manufacturer’s instructions, and additional PCR cycles were added where input DNA was less than the recommended 100ng according to the following equation (1):

(1) N = (100 / g) +7

where N = number of cycles and g = the input DNA in nanograms. N was rounded to the nearest integer.

Target Enrichment

Barley

The capture design targeted 31 barley genes that have known or suggested roles in important traits including frost resistance, vernalisation, row number, spike architecture, seed protein content and disease resistance. Additionally, 3000 SNPs derived from the commercially available Barley BOPA1 &2 SNP chips ^7^ were included. The enrichment was carried out with a custom-designed MYbaits kit (MYcroarray, Ann Arbor). A total of 25029 (80-mer length, 4 x flexible tiling density) biotinylated RNA probes were designed by Mycroarray. We used approx. 500 ng of DNA per capture reaction and used the lower annealing temperature (55**°**C) recommended for hybridization of ancient DNA (Mybaits protocol v. 3.0). Capture products were cleaned and quantified as per the library products.

Maize

This set of probes has previously been validated on ancient samples ^8^. Amplified library (150ng) was used in the capture reaction and the manufacturer’s standard hybridization temperature (65**°**C) was used. The washing steps and post-capture PCR were conducted following MYbaits protocol v. 2.3.1.

Rice

Details of the three enrichment methods. 1) The whole-genome (WG) approach was based on genomic DNA extracted from *O. sativa japonica* leaf tissue, which was converted to RNA baits. Target enrichment was performed using these baits according to the manufacturer’s instructions. 2) A custom MYbaits kit consisting of 18,985 baits (80-mer length, 4x tiling density) was designed from 70 domestication-associated loci known within the *O. sativa japonica* genome. Again, the protocol was performed according to the manufacturer’s instructions. 3) An enlarged bait design of 83,575 DNA baits (148 loci, 45-mer length 5x flexible tiling density) was set on a solid-state microarray chip compatible with Agilent gasket slides. Since no protocol exists for capture from these chips, we used the SureSelect (Agilent) protocol as a guide and followed it according to those instructions. Captured libraries were re-amplified with 15 cycles with Illumina-supplied primers under otherwise identical conditions to initial library construction.

Sequencing

Barley library and capture products were quantified using a Bioanalyzer and/or qPCR, and subsequently pooled at equimolar ratios. The ss libraries were sequenced on an Illumina MiSeq (75 bp, paired-end) and the ds libraries were sequenced on an Illumina Hiseq 2500 (125 bp paired-end) at the Norwegian Sequencing Centre. Grape and maize libraries were quantified with a TapeStation 2200 (Agilent) and pooled at equimolar ratios and were sequenced at the Danish National High-throughput DNA Sequencing Centre on an Illumina HiSeq 2500 platform (100 or 81 bp single read). The extraction blanks from the barley experiments were also sequenced at the Danish National High-throughput DNA Sequencing Centre. Libraries from grape, maize and barley extraction blanks were sequenced in different pools that also included other libraries from other taxa and non-charred grape and maize. Rice libraries were quantified using Qubit and Bioanalyzer and pooled at equimolar ratios. For each iteration of capture, libraries were sequenced at the University of Warwick on an Illumina MiSeq (250 bp single-end).

**Supplementary Figures**


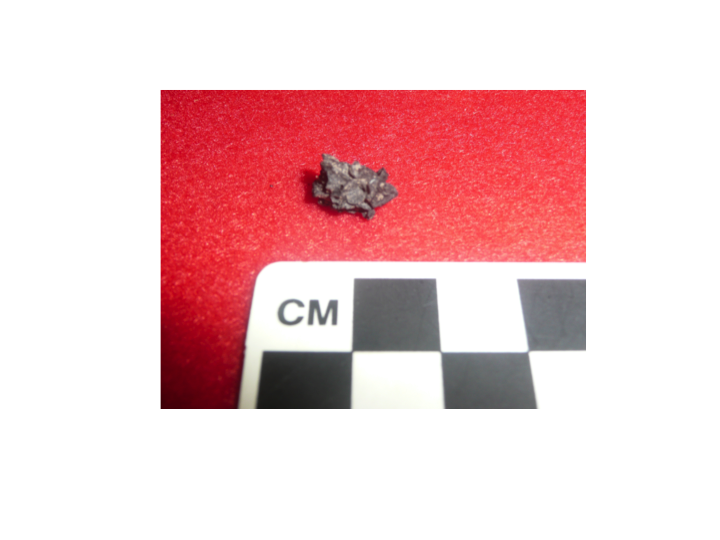


Figure S1. The lightly charred Montoya maize sample, excavated in 2011 by Karl Laumbach, dated to circa 3925 calibrated YBP that yielded 37 (shotgun) and 82 (capture) reads following PIA filtering. Further analysis of these reads showed only 26 to be authentic.

Figure S2. Representative examples of aDNA fragmentation and mis-incorporation patterns calculated using MapDamage v. 2.0.6. Note the damage only appears when mapping reads to the grape genome. The different panels show (a) Maize2 mapped to the grape genome, (b) Maize2 mapped to the maize genome, (c) Grape5 mapped to the grape genome, (d) Grape5 mapped to the maize genome, (e) Blank_barley1 mapped to the grape genome, and (f) Blank_barley2 mapped to the barley genome.

Figure S3. Schematic overview of the sample bleeding analysis. A) Dense clustering can lead to incorrect sequence assignment of indexes. B) Several types of reads are generated during single-index sequencing on the Illumina platform. Specific index cycles are used to read the sample-specific index in the P7 adapter. C) In cases where the DNA insert is longer than the sequencing mode the adapter and its index are not observed in the obtained sequencing read. D). In cases where the DNA insert is sufficiently short (i.e., in our case the number of sequencing cycles minus 40), the index can also be directly observed in the sequencing data itself. This allows for the assessment of mismatches between index read, and the index as observed in the sequencing data.

Table S1. Sample sequencing characteristics; the number of reads post adapter removal; the number of reads mapping to the target genome prior to duplicate removal; percentage clonality. All raw data is available on ENA (accession no. PRJEB15180).

| sample | Library name | #reads post adapter removal | #reads mapped | #reads mapped after duplicate removal | | % clonality |
| --- | --- | --- | --- | --- | --- | --- |
| Barley1a | VBA021E1L2i1_C1P1_S1_L001_R1_001 | 9304145 | 1255 | | 765 | 39.0 |
| Barley1b | 3-VBA021E1L1i1C1_S3_R1_001 | 2925924 | 1037 | | 839 | 19.1 |
| Barley2a | VBA022E1L2i1_C1P1_S2_L001_R1_001 | 6897638 | 555 | | 471 | 15.1 |
| Barley2b | 4-VBA022E1L1i1C1_S4_R1_001 | 3249226 | 1522 | | 1312 | 13.8 |
| Barley3a | VBA023E1L2_S11_L001_R1_001 | 1430106 | 142 | | 142 | 0.0 |
| Barley3b | 6-VBA023E1L1i1_S6_R1_001 | 4106051 | 894 | | 888 | 0.7 |
| Barley4a | VBA029E1L1i2_S10_L001_R1_001 | 6154074 | 514 | | 502 | 2.3 |
| Barley4b | 8-VBA029E1L2i2_S8_R1_001 | 243092 | 35 | | 34 | 2.9 |
| Barley5 | 1-VBA011E1L1i1C1_S1_R1_001 | 2605723 | 3405 | | 3211 | 5.7 |
| Barley6 | 2-VBA012E1L1i1C1_S2_R1_001 | 2357677 | 2237 | | 2004 | 10.4 |
| Barley7 | 5-VBA020E1L1i1_S5_R1_001 | 1745180 | 1347 | | 1332 | 1.1 |
| Barley8 | 7-VBA027E1L2i2_S7_R1_001 | 411863 | 60 | | 60 | 0.0 |
| Grape1 | TOG_K4RL_SA8796 | 6273171 | 168 | | 163 | 3.0 |
| Grape2 | TOG_K4RL_SA6123 | 8577411 | 241 | | 222 | 7.9 |
| Grape3 | TOG_Turkey_grape_SA_1370 | 6451116 | 498 | | 489 | 1.8 |
| Grape4 | TOG_Turkey_grape_SA_545 | 9040948 | 888 | | 838 | 5.6 |
| Grape5 | TOG_Turkey_grape_SA_5885 | 5746800 | 903 | | 881 | 2.4 |
| Maize1 | TOG_SYKL_Non_Grid_4_maize_MA_98 | 5023736 | 315 | | 314 | 0.3 |
| Maize2 | TOG_SYKL_Non_Grid_4_maize_MA_131 | 7315930 | 505 | | 504 | 0.2 |
| Maize3 | TOG_SYKL_Chinampas_maize_MA_87_ACAGAG_L008_R1_001 | 134236 | 27 | | 15 | 44.4 |
| Maize4 | TOG_SYKL_Barton_Creek_Cave_maize_1g | 10493365 | 1677 | | 1321 | 21.2 |
| Maize5 | TOG_SYKL_Barton_Creek_Cave_maize_1b | 7528231 | 1840 | | 1173 | 36.3 |
| Maize6 | TOG_SYKL_Barton_Creek_Cave_maize_1a | 7496187 | 1083 | | 892 | 17.6 |
| Maize7 | Sample_TOG_SYKL_Non_Grid_4_maize_MA_130 | 6548786 | 481 | | 475 | 1.2 |
| Maize8a | TOG_QCVT_Montoyo_2011_262 | 27977535 | 7270 | | 7191 | 1.1 |
| Maize8b | TOG_PKNF_Montoyo_2011_262_CAP | 21785491 | 7329 | | 3551 | 51.5 |
| Rice1 | Terr_S5_L001_R1_001 | 390515 | 33 | | 20 | 39.4 |
| Rice2 | Terr_trim | 500538 | 758 | | 73 | 90.4 |
| Rice3 | Balathal_S5_L001_R1_001 | 1783367 | 14 | | 12 | 14.3 |
| Rice4 | Balathal_S6_L001_R1_001 | 499372 | 7 | | 6 | 14.3 |
| Rice5 | BNW_0405_S6_L001_R1_001 | 4953182 | 68 | | 53 | 22.1 |
| Rice6 | BNW_0405_S7_L001_R1_001 | 567506 | 10 | | 9 | 10.0 |
| Rice7 | BNW405_S3_L001_R1_001 | 6101994 | 61 | | 52 | 14.8 |
| Rice8 | BNW_v200_S4_L001_R1_001 | 447690 | 19 | | 8 | 57.9 |
| Rice9 | NBJ214_S8_L001_R1_001 | 301980 | 7 | | 7 | 0.0 |
| Rice10 | NBJ219_S1_L001_R1_001 | 3425508 | 16 | | 11 | 31.3 |
| Rice11 | NUL105_S3_L001_R1_001 | 592182 | 135 | | 78 | 42.2 |
| Rice12a | Old_Sima_precapture_S1_L001_R1_001 | 2608916 | 0 | | 0 | NA |
| Rice12b | Old_Sima_S2_L001_R1_001 | 1476971 | 24 | | 16 | 33.3 |
| Rice13 | Balathal_trim | 396830 | 707 | | 49 | 93.1 |
| Rice14 | BNW_v200_trim | 1096766 | 1374 | | 199 | 85.5 |
| Rice15 | NBJ_214_trim | 595556 | 652 | | 36 | 94.5 |
| Rice16 | NUL_105_trim | 338135 | 486 | | 29 | 94.0 |
| Rice17 | Old_Sima_trim | 373420 | 51 | | 6 | 88.2 |
| Blank_barley1 | TOG_SYKL_HN034NCL1_barley_ext_NC | 32461241 | NA | | NA | NA |
| Blank_barley2 | TOG_SYKL_HN018NCL1_barley_ext_NC | 6865758 | NA | | NA | NA |
| Blank_barley3 | TOG_SYKL_HN002NCL1_barley_ext_NC | 602109 | NA | | NA | NA |
| Blank_grape1 | TOG_K4RL_Extraction_blank_2_TGATCG_L006_R1_001 | 31965087 | NA | | NA | NA |
| Blank_maize1 | TOG_QCVT_Extraction_blank | 41391139 | NA | | NA | NA |
| Blank_maize2 | TOG_SYKL_Extraction_blank_maize | 15005456 | NA | | NA | NA |
| Blank_rice1 | Rice-blank | 250044 | NA | | NA | NA |

Table S2. The number and percentage of reads mapping to each of the four genomes used in this study. *indicates that typical aDNA damage patterns were observed.

| sample | Barley | % Barley | Grape | % Grape | Maize | %Maize | Rice | %Rice |
| --- | --- | --- | --- | --- | --- | --- | --- | --- |
| Barley1a | 765 | 0.008 | 490 | 0.005 | 647 | 0.007 | 639 | 0.007 |
| Barley1b | 839 | 0.029 | 593 | 0.020 | 684 | 0.023 | 618 | 0.021 |
| Barley2a | 471 | 0.007 | 248 | 0.004 | 387 | 0.006 | 347 | 0.005 |
| Barley2b | 1312 | 0.040 | 736 | 0.023 | 935 | 0.029 | 932 | 0.029 |
| Barley3a | 142 | 0.010 | 23 | 0.002 | 90 | 0.006 | 75 | 0.005 |
| Barley3b | 888 | 0.022 | 199 | 0.005 | 648 | 0.016 | 507 | 0.012 |
| Barley4a | 502 | 0.008 | 101 | 0.002 | 386 | 0.006 | 319 | 0.005 |
| Barley4b | 34 | 0.014 | 8 | 0.003 | 9 | 0.004 | 9 | 0.004 |
| Barley5 | 3211 | 0.123 | 1884 | 0.072 | 2144 | 0.082 | 1877 | 0.072 |
| Barley6 | 2004 | 0.085 | 958 | 0.041 | 1465 | 0.062 | 1416 | 0.060 |
| Barley7 | 1332 | 0.076 | 811 | 0.046 | 801 | 0.046 | 651 | 0.037 |
| Barley8 | 60 | 0.015 | 21 | 0.005 | 50 | 0.012 | 32 | 0.008 |
| Grape1 | 1539 | 0.025 | 163 | 0.003 | 1151 | 0.018 | 1048 | 0.017 |
| Grape2 | 1976 | 0.023 | 222 | 0.003 | 1870 | 0.022 | 1766 | 0.021 |
| Grape3 | 349 | 0.005 | 489* | 0.008 | 577 | 0.009 | 204 | 0.003 |
| Grape4 | 1252 | 0.014 | 838* | 0.009 | 3860 | 0.043 | 540 | 0.006 |
| Grape5 | 1349 | 0.023 | 881* | 0.015 | 762 | 0.013 | 652 | 0.011 |
| Maize1 | 379 | 0.008 | 490* | 0.010 | 314 | 0.006 | 288 | 0.006 |
| Maize2 | 527 | 0.007 | 482* | 0.007 | 504 | 0.007 | 511 | 0.007 |
| Maize3 | 11 | 0.008 | 24 | 0.018 | 15 | 0.011 | 4 | 0.003 |
| Maize4 | 2543 | 0.024 | 1151* | 0.011 | 1321 | 0.013 | 779 | 0.007 |
| Maize5 | 2301 | 0.031 | 805* | 0.011 | 1173 | 0.016 | 486 | 0.006 |
| Maize6 | 1659 | 0.022 | 709* | 0.009 | 892 | 0.012 | 464 | 0.006 |
| Maize7 | 599 | 0.009 | 458* | 0.007 | 475 | 0.007 | 474 | 0.007 |
| Maize8a | 9346 | 0.033 | 3144* | 0.011 | 7191 | 0.026 | 6078 | 0.022 |
| Maize8b | 4412 | 0.020 | 3262* | 0.015 | 3551 | 0.016 | 3136 | 0.014 |
| Rice1 | 78 | 0.020 | 31 | 0.008 | 17 | 0.004 | 20 | 0.005 |
| Rice2 | 86 | 0.017 | 31 | 0.006 | 77 | 0.015 | 73 | 0.015 |
| Rice3 | 30 | 0.002 | 14 | 0.001 | 14 | 0.001 | 12 | 0.001 |
| Rice4 | 27 | 0.005 | 43 | 0.009 | 3 | 0.001 | 6 | 0.001 |
| Rice5 | 75 | 0.002 | 69 | 0.001 | 63 | 0.001 | 53 | 0.001 |
| Rice6 | 13 | 0.002 | 52 | 0.009 | 5 | 0.001 | 9 | 0.002 |
| Rice7 | 61 | 0.001 | 53 | 0.001 | 34 | 0.001 | 52 | 0.001 |
| Rice8 | 10 | 0.002 | 37 | 0.008 | 5 | 0.001 | 8 | 0.002 |
| Rice9 | 5 | 0.002 | 8 | 0.003 | 2 | 0.001 | 7 | 0.002 |
| Rice10 | 33 | 0.001 | 13 | 0.000 | 16 | 0.000 | 11 | 0.000 |
| Rice11 | 357 | 0.060 | 221 | 0.037 | 55 | 0.009 | 78 | 0.013 |
| Rice12a | 1 | 0.000 | 1 | 0.000 | 0 | 0.000 | 0 | 0.000 |
| Rice12b | 41 | 0.003 | 106 | 0.007 | 12 | 0.001 | 16 | 0.001 |
| Rice13 | 43 | 0.011 | 24 | 0.006 | 40 | 0.010 | 49 | 0.012 |
| Rice14 | 195 | 0.018 | 54 | 0.005 | 177 | 0.016 | 199 | 0.018 |
| Rice15 | 40 | 0.007 | 14 | 0.002 | 34 | 0.006 | 36 | 0.006 |
| Rice16 | 82 | 0.024 | 82 | 0.024 | 15 | 0.004 | 29 | 0.009 |
| Rice17 | 26 | 0.007 | 26 | 0.007 | 9 | 0.002 | 6 | 0.002 |
| Blank_barley1 | 713 | 0.002 | 106* | 0.000 | 432 | 0.001 | 91 | 0.000 |
| Blank_barley2 | 5398 | 0.013 | 1902* | 0.005 | 4055 | 0.010 | 1476 | 0.004 |
| Blank_barley3 | 592 | 0.002 | 964* | 0.003 | 395 | 0.001 | 269 | 0.001 |
| Blank_grape1 | 1624 | 0.024 | 1565 | 0.023 | 1133 | 0.017 | 587 | 0.009 |
| Blank_maize1 | 1833 | 0.304 | 1338 | 0.222 | 1400 | 0.233 | 1078 | 0.179 |
| Blank_maize2 | 2003 | 0.013 | 747* | 0.005 | 1125 | 0.007 | 531 | 0.004 |
| Blank_rice1 | 0 | 0.000 | 0 | 0.000 | 0 | 0.000 | 0 | 0.000 |

Table S3. Proportion of bled indices (conflicting index identity observed at the end of the unfiltered sequencing read) in the 15 libraries showing aDNA damage patterns when mapped to the grape genome. The proportion of bled indexes from non-charred grape samples (not part of this study) with at least 10% endogenous aDNA (bled grape indexes) was quantified and compared to that of samples from other species sequenced on the same lane (non-grape indexes). We observed significantly more bled indexes in those reads aligned to the grape genome only compared to those in the overall, pre-aligned sequencing data (Wilcoxon Signed Rank, *W* = 0, *N* = 15, *p* < 0.05). We also observed significantly more bled indexes from bled non-charred grape samples compared to those indexes from non-grape samples sequenced in the same pool as the charred grape, charred maize and extraction blanks (Wilcoxon Signed Rank, *W* = 0, *N* = 15, *p* < 0.05).

|  |  |  | Bled grape indexes (%) | |  | Bled non-grape indexes (%) | |
| --- | --- | --- | --- | --- | --- | --- | --- |
| Samples | Index | Pool | Overall | Aligned  to grape |  | Overall | Aligned  to grape |
| TOG_SYKL_Non_Grid_4_maize_MA_130 | CGTATA | 1* | 0.02 | 22 |  | 0.16 | 1.4 |
| TOG_SYKL_Barton_Creek_Cave_maize_1a | CGTAGT | 1 | 0.01 | 7 |  | 0.37 | 1.2 |
| TOG_SYKL_Barton_Creek_Cave_maize_1b | CAGCAC | 1 | 0.01 | 8 |  | 0.11 | 0.0 |
| TOG_SYKL_Barton_Creek_Cave_maize_1g | ACTGCG | 1 | 0.01 | 4 |  | 0.11 | 0.0 |
| TOG_SYKL_Extraction_blank_maize | TCTCGC | 1 | 0.01 | 6 |  | 0.13 | 0.4 |
| TOG_SYKL_HN002NCL1_barley_ext_NC | AACTCC | 1 | 0.00 | 10 |  | 0.03 | 0.3 |
| TOG_SYKL_HN018NCL1_barley_ext_NC | TTGAAG | 1 | 0.00 | 15 |  | 0.10 | 1.1 |
| TOG_SYKL_HN034NCL1_barley_ext_NC | ACTATC | 1 | 0.00 | 26 |  | 0.04 | 0.7 |
| TOG_SYKL_Non_Grid_4_maize_MA_131 | TGATCG | 1 | 0.04 | 38 |  | 0.31 | 1.5 |
| TOG_SYKL_Non_Grid_4_maize_MA_98 | CTATCA | 1 | 0.03 | 39 |  | 0.28 | 0.0 |
| TOG_Turkey_grape_SA_1370 | ACATAC | 1 | 0.03 | 24 |  | 0.30 | 0.0 |
| TOG_Turkey_grape_SA_545 | CAGCTA | 1 | 0.01 | 14 |  | 0.12 | 0.0 |
| TOG_Turkey_grape_SA_5885 | TGAGCC | 1 | 0.01 | 11 |  | 0.10 | 1.2 |
| TOG_PKNF_Montoyo_2011_262_CAP | GTGTAT | 2# | 0.09 | 7 |  | 0.06 | 0.1 |
| TOG_QCVT_Montoyo_2011_262 | GTGTAT | 3§ | 0.05 | 4 |  | 0.17 | 2.4 |

* Indexes of ancient non-charred grape samples with > 10% endogenous DNA – AGCATG, CGATGA, TGCATA and TGTGAC. Indexes of non-grape samples – CATAGA and TGACGT.

# Indexes of ancient non-charred grape samples with > 10% endogenous DNA – TGCAGG, GAGTAG, AGTGAG, CTCTGC TGTGAC, TGCATA and TCTCGC. Indexes of non-grape samples – CGCTAT, TGAACA, TGAGCC, CGATGA, TGATGC, GACGAC and TCTATG.

§ Indexes of ancient non-charred grape samples with > 10% endogenous DNA – CGTAGT, TCGATG, CTATCA, CGTATA, TGATCG, ACTGCG, and GTCGTC. Indexes of non-grape samples – TGAGCC, AGCATG, CGATGA, TGATGC, GACGAC, TGAACA, and GTATCT.

Table S4. Percentage taxonomic assignment of reads from each sample determined using MEGAN. Results indicate assignment prior to PIA filtering.

|  | **Barley1a** | **Barley1b** | **Barley2a** | **Barley2b** | **Barley3a** | **Barley3b** |  |
| --- | --- | --- | --- | --- | --- | --- | --- |
| **Bacteria** | 75.99 | 77.90 | 72.43 | 77.89 | 74.96 | 74.16 |  |
| **Eukaryotes** | 2.13 | 1.98 | 1.89 | 1.85 | 0.68 | 3.55 |  |
| **Plants** | 0.62 | 0.36 | 0.56 | 0.58 | 0.41 | 0.67 |  |
| **Target** | 0.02 | 0.01 | 0.02 | 0.00 | 0.02 | 0.02 |  |
| **Not assigned** | 21.24 | 19.75 | 25.10 | 19.67 | 23.94 | 21.60 |  |
|  | **Barley4a** | **Barley4b** | **Barley5** | **Barley6** | **Barley7** | **Barley8** |  |
| **Bacteria** | 69.31 | 71.05 | 76.18 | 75.88 | 80.01 | 67.43 |  |
| **Eukaryotes** | 0.86 | 3.48 | 2.72 | 1.35 | 1.26 | 0.83 |  |
| **Plants** | 0.43 | 0.79 | 0.37 | 0.42 | 0.28 | 0.53 |  |
| **Target** | 0.01 | 0.02 | 0.01 | 0.01 | 0.01 | 0.01 |  |
| **Not assigned** | 29.39 | 24.66 | 20.73 | 22.34 | 18.43 | 31.20 |  |
|  | **Grape1** | **Grape2** | **Grape3** | **Grape4** | **Grape5** |  |  |
| **Bacteria** | 71.79 | 76.78 | 75.23 | 67.37 | 70.16 |  |  |
| **Eukaryotes** | 6.40 | 2.34 | 2.45 | 7.27 | 4.61 |  |  |
| **Plants** | 1.00 | 2.42 | 1.06 | 2.80 | 1.94 |  |  |
| **Target** | 0.02 | 0.01 | 0.28 | 0.21 | 0.34 |  |  |
| **Not assigned** | 20.79 | 18.46 | 20.97 | 22.36 | 22.95 |  |  |
|  | **Maize1** | **Maize2** | **Maize3** | **Maize4** | **Maize5** | **Maize6** |  |
| **Bacteria** | 78.43 | 80.80 | 71.75 | 61.16 | 54.25 | 61.75 |  |
| **Eukaryotes** | 1.14 | 0.60 | 2.72 | 10.49 | 19.59 | 12.17 |  |
| **Plants** | 0.89 | 0.56 | 1.95 | 0.51 | 0.65 | 0.45 |  |
| **Target** | 0.00 | 0.00 | 0.00 | 0.01 | 0.02 | 0.01 |  |
| **Not assigned** | 19.54 | 18.05 | 23.58 | 27.83 | 25.49 | 25.62 |  |
|  | **Maize7** | **Maize8a** | **Maize8b** |  |  |  |  |
| **Bacteria** | 78.01 | 72.55 | 67.49 |  |  |  |  |
| **Eukaryotes** | 1.03 | 4.04 | 6.89 |  |  |  |  |
| **Plants** | 0.78 | 2.07 | 4.32 |  |  |  |  |
| **Target** | 0.00 | 0.26 | 0.38 |  |  |  |  |
| **Not assigned** | 20.17 | 21.08 | 20.92 |  |  |  |  |
|  | **Rice1** | **Rice2** | **Rice3** | **Rice4** | **Rice5** | **Rice6** |  |
| **Bacteria** | 88.69 | 61.34 | 13.29 | 87.52 | 22.33 | 49.20 |  |
| **Eukaryotes** | 0.72 | 9.70 | 0.47 | 0.84 | 0.54 | 1.28 |  |
| **Plants** | 0.83 | 4.71 | 0.11 | 0.49 | 0.19 | 0.69 |  |
| **Target** | 0.03 | 0.00 | 0.00 | 0.04 | 0.03 | 0.00 |  |
| **Not assigned** | 9.73 | 24.25 | 86.14 | 11.11 | 76.91 | 48.82 |  |
|  | **Rice7** | **Rice8** | **Rice9** | **Rice10** | **Rice11** | **Rice12a** | **Rice12b** |
| **Bacteria** | 16.33 | 89.63 | 85.93 | 8.62 | 74.74 | 59.33 | 63.79 |
| **Eukaryotes** | 2.90 | 1.25 | 0.39 | 0.69 | 4.78 | 0.71 | 1.21 |
| **Plants** | 0.13 | 0.41 | 0.03 | 0.12 | 0.73 | 0.21 | 0.15 |
| **Target** | 0.02 | 0.00 | 0.00 | 0.01 | 0.03 | 0.03 | 0.02 |
| **Not assigned** | 80.63 | 8.70 | 13.65 | 90.57 | 19.72 | 39.71 | 34.83 |
|  | **Rice13** | **Rice14** | **Rice15** | **Rice16** | **Rice17** |  |  |
| **Bacteria** | 64.87 | 61.54 | 69.16 | 61.54 | 54.09 |  |  |
| **Eukaryotes** | 7.85 | 11.19 | 5.67 | 11.19 | 8.07 |  |  |
| **Plants** | 3.26 | 4.02 | 2.24 | 4.02 | 2.62 |  |  |
| **Target** | 1.18 | 0.29 | 0.60 | 0.29 | 0.18 |  |  |
| **Not assigned** | 22.84 | 22.96 | 22.32 | 22.96 | 35.03 |  |  |
|  | **Blank_bar1** | **Blank_bar2** | **Blank_bar3** | **Blank_gra1** | **Blank_mai1** | **Blank_mai2** | **Blank_ric1** |
| **Bacteria** | 23.72 | 15.21 | 34.86 | 5.21 | 6.62 | 59.93 | 0.58 |
| **Eukaryotes** | 5.97 | 13.88 | 2.14 | 9.74 | 4.95 | 15.19 | 0.39 |
| **Plants** | 1.08 | 0.82 | 0.76 | 0.18 | 0.17 | 0.58 | 0.19 |
| **Target** | 0.02 | 0.00 | 0.01 | 0.01 | 0.00 | 0.01 | 0.00 |
| **Not assigned** | 69.22 | 70.08 | 62.24 | 84.86 | 88.25 | 24.29 | 98.84 |

Table S5. Percent of reads (496 total) from Bunning et al., (2012)^9^ assigned to different taxonomic groups using BLASTn and MEGAN. No PIA filtering was performed.

| **Taxonomic group** | **Reads (%)** |
| --- | --- |
| **eukaryotes** | **20.1** |
| *Mus musculus* | 4.7 |
| *Zea mays* | 0.2 |
| *Hordeum vulgare* | 0.2 |
| **bacteria** | **0.2** |
| **not assigned** | **2.9** |
| **no hits** | **76.8** |

Table S6. Output from RepeatMasker identifying reads that contain regions of simple repeats or low complexity in the data (496 reads) from Bunning et al (2012)^9^.

| query | position | position | query | matching | repeat |  |
| --- | --- | --- | --- | --- | --- | --- |
| sequence | begin | end | (left) | repeat | class/family | ID |
|  |  |  |  |  |  |  |
| 10048486-1 | 22 | 50 | (0) + | (TCCCT)n | Simple_repeat | 1 |
| 10152843-1 | 21 | 48 | (2) + | (TTTGG)n | Simple_repeat | 2 |
| 10152843-1_1 | 21 | 48 | (2) + | (TTTGG)n | Simple_repeat | 3 |
| 1016967-2 | 2 | 27 | (23) + | (ATCGGG)n | Simple_repeat | 4 |
| 10294390-1 | 20 | 47 | (3) + | (TTACA)n | Simple_repeat | 5 |
| 1031175-2 | 26 | 50 | (0) + | (GGTTT)n | Simple_repeat | 6 |
| 10431453-1 | 20 | 48 | (2) + | (AGGGA)n | Simple_repeat | 7 |
| 10601560-1 | 12 | 45 | (5) + | (GAAGG)n | Simple_repeat | 8 |
| 10813343-1 | 23 | 48 | (2) + | (CGTCT)n | Simple_repeat | 9 |
| 1088949-2 | 25 | 50 | (0) + | (TGGTT)n | Simple_repeat | 10 |
| 1100487-2 | 25 | 50 | (0) + | (TGGTT)n | Simple_repeat | 11 |
| 11040810-1 | 19 | 48 | (2) + | (GATCA)n | Simple_repeat | 12 |
| 11067821-1 | 21 | 47 | (3) + | (TTTGT)n | Simple_repeat | 13 |
| 11228407-1 | 8 | 41 | (9) + | (ATTCC)n | Simple_repeat | 14 |
| 11238018-1 | 21 | 50 | (0) + | (TTTGG)n | Simple_repeat | 15 |
| 11325380-1 | 1 | 48 | (2) + | (TTCGT)n | Simple_repeat | 16 |
| 11345071-1 | 4 | 44 | (6) + | (GTTTC)n | Simple_repeat | 17 |
| 1135089-2 | 21 | 50 | (0) + | (TTTGG)n | Simple_repeat | 18 |
| 11390245-1 | 21 | 46 | (4) + | (TTTGG)n | Simple_repeat | 19 |
| 11607146-1 | 19 | 45 | (5) + | (GTTTC)n | Simple_repeat | 20 |
| 1164295-2 | 21 | 50 | (0) + | (TTTGG)n | Simple_repeat | 21 |
| 11654590-1 | 18 | 45 | (5) + | (TCTAT)n | Simple_repeat | 22 |
| 11689919-1 | 20 | 46 | (4) + | (TTATA)n | Simple_repeat | 23 |
| 11720167-1 | 7 | 31 | (19) + | (GAGGTC)n | Simple_repeat | 24 |
| 1182417-2 | 21 | 50 | (0) + | (GTAGG)n | Simple_repeat | 25 |
| 11846174-1 | 21 | 49 | (1) + | (TTTGG)n | Simple_repeat | 26 |
| 11969090-1 | 13 | 47 | (3) + | (AGATG)n | Simple_repeat | 27 |
| 1197270-2 | 17 | 49 | (1) + | (TTGGT)n | Simple_repeat | 28 |
| 12254921-1 | 21 | 49 | (1) + | (TTTGG)n | Simple_repeat | 29 |
| 12330456-1 | 17 | 47 | (3) + | (GGTGC)n | Simple_repeat | 30 |
| 12337936-1 | 19 | 50 | (0) + | (GGTTT)n | Simple_repeat | 31 |
| 12579377-1 | 10 | 37 | (13) + | (TTCCT)n | Simple_repeat | 32 |
| 12647283-1 | 25 | 50 | (0) + | (TGGTT)n | Simple_repeat | 33 |
| 12703697-1 | 25 | 50 | (0) + | (TGGTT)n | Simple_repeat | 34 |
| 12785579-1 | 16 | 43 | (7) + | (TTCCT)n | Simple_repeat | 35 |
| 12904317-1 | 25 | 50 | (0) + | (TGGTT)n | Simple_repeat | 36 |
| 12962522-1 | 1 | 29 | (21) + | (CAAGG)n | Simple_repeat | 37 |
| 13029551-1 | 21 | 47 | (3) + | (TTGGG)n | Simple_repeat | 38 |
| 13220067-1 | 25 | 50 | (0) + | (GTTGG)n | Simple_repeat | 39 |
| 13247855-1 | 21 | 48 | (2) + | (AAGGG)n | Simple_repeat | 40 |
| 13443631-1 | 2 | 22 | (28) + | (AAAC)n | Simple_repeat | 41 |
| 1345349-1 | 25 | 50 | (0) + | (TGGTT)n | Simple_repeat | 42 |
| 1347778-1 | 25 | 49 | (1) + | (GTTTG)n | Simple_repeat | 43 |
| 1347778-1_1 | 25 | 49 | (1) + | (GTTTG)n | Simple_repeat | 44 |
| 13623661-1 | 22 | 50 | (0) + | (TTTGG)n | Simple_repeat | 45 |
| 13633601-1 | 17 | 45 | (5) + | (TTTAG)n | Simple_repeat | 46 |
| 13714095-1 | 27 | 48 | (2) + | (TTCC)n | Simple_repeat | 47 |
| 13735176-1 | 8 | 41 | (9) + | (CCTTG)n | Simple_repeat | 48 |
| 13892964-1 | 22 | 50 | (0) + | (TTTAT)n | Simple_repeat | 49 |
| 1396398-1 | 25 | 50 | (0) + | (CGGTT)n | Simple_repeat | 50 |
| 14120764-1 | 15 | 48 | (2) + | (CGTTG)n | Simple_repeat | 51 |
| 1429037-1 | 17 | 50 | (0) + | (GCGTG)n | Simple_repeat | 52 |
| 14375115-1 | 24 | 50 | (0) + | (GTTTG)n | Simple_repeat | 53 |
| 14407538-1 | 30 | 50 | (0) + | (AACC)n | Simple_repeat | 54 |
| 1444007-1 | 5 | 50 | (0) + | (TTCGT)n | Simple_repeat | 55 |
| 14465358-1 | 17 | 45 | (5) + | (TAATT)n | Simple_repeat | 56 |
| 14526380-1 | 16 | 50 | (0) + | (TAGAA)n | Simple_repeat | 57 |
| 14547569-1 | 17 | 50 | (0) + | (TTGGT)n | Simple_repeat | 58 |
| 14701649-1 | 22 | 50 | (0) + | (TTTTG)n | Simple_repeat | 59 |
| 148927-10 | 25 | 50 | (0) + | (TGGTT)n | Simple_repeat | 60 |
| 15261-60 | 25 | 50 | (0) + | (TGGTT)n | Simple_repeat | 61 |
| 15326168-1 | 22 | 50 | (0) + | (TTTTG)n | Simple_repeat | 62 |
| 15340271-1 | 16 | 43 | (7) + | (GTTTT)n | Simple_repeat | 63 |
| 15350729-1 | 20 | 50 | (0) + | (CTTTA)n | Simple_repeat | 64 |
| 15357041-1 | 17 | 49 | (1) + | (TCGTT)n | Simple_repeat | 65 |
| 15513468-1 | 26 | 50 | (0) + | (GTTTG)n | Simple_repeat | 66 |
| 15881599-1 | 18 | 48 | (2) + | (AGATC)n | Simple_repeat | 67 |
| 15957733-1 | 7 | 50 | (0) + | (TCGTC)n | Simple_repeat | 68 |
| 16027021-1 | 17 | 49 | (1) + | (TTGGT)n | Simple_repeat | 69 |
| 16103647-1 | 21 | 49 | (1) + | (TTTGG)n | Simple_repeat | 70 |
| 16268688-1 | 16 | 45 | (5) + | (TTCCC)n | Simple_repeat | 71 |
| 1627378-1 | 17 | 48 | (2) + | (TTGTT)n | Simple_repeat | 72 |
| 16340727-1 | 21 | 50 | (0) + | (TTTGG)n | Simple_repeat | 73 |
| 16380715-1 | 24 | 50 | (0) + | (GTTTG)n | Simple_repeat | 74 |
| 16380715-1_1 | 24 | 50 | (0) + | (GTTTG)n | Simple_repeat | 75 |
| 16481102-1 | 17 | 50 | (0) + | (TTGTT)n | Simple_repeat | 76 |
| 16528290-1 | 21 | 50 | (0) + | (GTAGG)n | Simple_repeat | 77 |
| 16936800-1 | 25 | 49 | (1) + | (TTACA)n | Simple_repeat | 78 |
| 16936800-1_1 | 25 | 49 | (1) + | (TTACA)n | Simple_repeat | 79 |
| 16959280-1 | 26 | 50 | (0) + | (TGCCT)n | Simple_repeat | 80 |
| 16990753-1 | 6 | 44 | (6) + | (TGGAGC)n | Simple_repeat | 81 |
| 17034223-1 | 17 | 49 | (1) + | (TTGTT)n | Simple_repeat | 82 |
| 17068630-1 | 11 | 45 | (5) + | (ATCGT)n | Simple_repeat | 83 |
| 17362101-1 | 19 | 50 | (0) + | G-rich | Low_complexity | 84 |
| 17388027-1 | 21 | 47 | (3) + | (TTGGG)n | Simple_repeat | 85 |
| 17415765-1 | 22 | 48 | (2) + | (AGAAT)n | Simple_repeat | 86 |
| 1778446-1 | 3 | 34 | (16) + | (CCAA)n | Simple_repeat | 87 |
| 18168520-1 | 17 | 42 | (8) + | (GCGGG)n | Simple_repeat | 88 |
| 18168520-1_1 | 17 | 42 | (8) + | (GCGGG)n | Simple_repeat | 89 |
| 18216687-1 | 1 | 44 | (6) + | GA-rich | Low_complexity | 90 |
| 18231519-1 | 25 | 50 | (0) + | (TGGTT)n | Simple_repeat | 91 |
| 18300380-1 | 3 | 41 | (9) + | (CCTTC)n | Simple_repeat | 92 |
| 18301610-1 | 15 | 40 | (10) + | (TTTTG)n | Simple_repeat | 93 |
| 18387644-1 | 1 | 41 | (9) + | (TTCCT)n | Simple_repeat | 94 |
| 18474286-1 | 25 | 50 | (0) + | (TGGTT)n | Simple_repeat | 95 |
| 18682238-1 | 20 | 46 | (4) + | (GGAAG)n | Simple_repeat | 96 |
| 18939921-1 | 21 | 50 | (0) + | (TTTGG)n | Simple_repeat | 97 |
| 18962224-1 | 21 | 47 | (3) + | (TTGGG)n | Simple_repeat | 98 |
| 18987132-1 | 21 | 50 | (0) + | (TTGGG)n | Simple_repeat | 99 |
| 19019884-1 | 21 | 49 | (1) + | (TTTGG)n | Simple_repeat | 100 |
| 19084428-1 | 22 | 47 | (3) + | (CTGAC)n | Simple_repeat | 101 |
| 1924445-1 | 11 | 27 | (23) + | (TTTTC)n | Simple_repeat | 102 |
| 1924445-1 | 28 | 49 | (1) + | (T)n | Simple_repeat | 103 |
| 1925729-1 | 2 | 27 | (23) + | (ATCGGG)n | Simple_repeat | 104 |
| 19481425-1 | 1 | 48 | (2) + | (TTCCT)n | Simple_repeat | 105 |
| 19722538-1 | 21 | 47 | (3) + | (TTGGG)n | Simple_repeat | 106 |
| 1972449-1 | 10 | 50 | (0) + | (TTGGT)n | Simple_repeat | 107 |
| 19783928-1 | 2 | 27 | (23) + | (GATCGG)n | Simple_repeat | 108 |
| 1978538-1 | 6 | 49 | (1) + | (GTTAG)n | Simple_repeat | 109 |
| 19817749-1 | 12 | 40 | (10) + | (TGAAT)n | Simple_repeat | 110 |
| 19847809-1 | 17 | 46 | (4) + | (GTTTG)n | Simple_repeat | 111 |
| 19856984-1 | 24 | 50 | (0) + | (CAAAC)n | Simple_repeat | 112 |
| 19859335-1 | 21 | 49 | (1) + | (TAATT)n | Simple_repeat | 113 |
| 19881523-1 | 21 | 48 | (2) + | (TTTTA)n | Simple_repeat | 114 |
| 20328585-1 | 1 | 42 | (8) + | GA-rich | Low_complexity | 115 |
| 20345823-1 | 26 | 50 | (0) + | (GTTTG)n | Simple_repeat | 116 |
| 20364960-1 | 25 | 50 | (0) + | (TGGTT)n | Simple_repeat | 117 |
| 20415854-1 | 26 | 50 | (0) + | (GCTTA)n | Simple_repeat | 118 |
| 20536884-1 | 21 | 47 | (3) + | (TTGGG)n | Simple_repeat | 119 |
| 20704038-1 | 22 | 49 | (1) + | (TTTTG)n | Simple_repeat | 120 |
| 20921607-1 | 25 | 50 | (0) + | (TGGTT)n | Simple_repeat | 121 |
| 2092274-1 | 2 | 35 | (15) + | (AATTC)n | Simple_repeat | 122 |
| 20953829-1 | 19 | 50 | (0) + | (TGTAC)n | Simple_repeat | 123 |
| 21122375-1 | 14 | 43 | (7) + | (GATGT)n | Simple_repeat | 124 |
| 23948-40 | 25 | 50 | (0) + | (TGGTT)n | Simple_repeat | 125 |
| 2604907-1 | 1 | 48 | (2) + | (TTCGT)n | Simple_repeat | 126 |
| 2671951-1 | 19 | 45 | (5) + | (TTCC)n | Simple_repeat | 127 |
| 2702668-1 | 21 | 47 | (3) + | (TTGGG)n | Simple_repeat | 128 |
| 2739699-1 | 2 | 22 | (28) + | (AAAC)n | Simple_repeat | 129 |
| 2901429-1 | 18 | 45 | (5) + | (CGTTT)n | Simple_repeat | 130 |
| 2915395-1 | 2 | 39 | (11) + | (ATATT)n | Simple_repeat | 131 |
| 2925145-1 | 21 | 50 | (0) + | (TTGGT)n | Simple_repeat | 132 |
| 294552-5 | 25 | 50 | (0) + | (TGGTT)n | Simple_repeat | 133 |
| 2960497-1 | 21 | 47 | (3) + | (TTGGG)n | Simple_repeat | 134 |
| 2975572-1 | 25 | 50 | (0) + | (TGGTT)n | Simple_repeat | 135 |
| 3017239-1 | 19 | 50 | (0) + | (CTTGC)n | Simple_repeat | 136 |
| 3138401-1 | 21 | 50 | (0) + | (TTTAT)n | Simple_repeat | 137 |
| 3233485-1 | 20 | 50 | (0) + | (CTTTC)n | Simple_repeat | 138 |
| 3292303-1 | 13 | 43 | (7) + | (ATGTT)n | Simple_repeat | 139 |
| 3433-219 | 25 | 50 | (0) + | (TGGTT)n | Simple_repeat | 140 |
| 3496867-1 | 25 | 50 | (0) + | (TTCCT)n | Simple_repeat | 141 |
| 357646-4 | 26 | 50 | (0) + | (GTTTG)n | Simple_repeat | 142 |
| 3714808-1 | 12 | 50 | (0) + | (GCCGT)n | Simple_repeat | 143 |
| 3868609-1 | 22 | 48 | (2) + | (TTTTG)n | Simple_repeat | 144 |
| 3886686-1 | 21 | 49 | (1) + | (TTTGG)n | Simple_repeat | 145 |
| 3953014-1 | 21 | 47 | (3) + | (TTACT)n | Simple_repeat | 146 |
| 3955536-1 | 1 | 50 | (0) + | (TTCGT)n | Simple_repeat | 147 |
| 4134876-1 | 17 | 38 | (12) + | (CATG)n | Simple_repeat | 148 |
| 4143378-1 | 14 | 39 | (11) + | (ACCAA)n | Simple_repeat | 149 |
| 416482-4 | 21 | 48 | (2) + | (TTTGG)n | Simple_repeat | 150 |
| 416482-4_1 | 21 | 48 | (2) + | (TTTGG)n | Simple_repeat | 151 |
| 416860-4 | 21 | 47 | (3) + | (TTGGG)n | Simple_repeat | 152 |
| 4186351-1 | 8 | 48 | (2) + | (CGTTT)n | Simple_repeat | 153 |
| 4432079-1 | 25 | 50 | (0) + | (TGGTT)n | Simple_repeat | 154 |
| 4440527-1 | 2 | 50 | (0) + | (CTTTT)n | Simple_repeat | 155 |
| 4460445-1 | 3 | 27 | (23) + | (TCGGGA)n | Simple_repeat | 156 |
| 4488081-1 | 18 | 48 | (2) + | (AGATC)n | Simple_repeat | 157 |
| 449564-4 | 26 | 50 | (0) + | (GTTTG)n | Simple_repeat | 158 |
| 453717-4 | 25 | 50 | (0) + | (CGGTT)n | Simple_repeat | 159 |
| 4653218-1 | 23 | 48 | (2) + | (AAGGC)n | Simple_repeat | 160 |
| 4655562-1 | 22 | 50 | (0) + | (TTAGG)n | Simple_repeat | 161 |
| 4726103-1 | 8 | 50 | (0) + | (CTTTC)n | Simple_repeat | 162 |
| 4746551-1 | 12 | 49 | (1) + | (TCCCT)n | Simple_repeat | 163 |
| 4769062-1 | 21 | 49 | (1) + | (TTTTG)n | Simple_repeat | 164 |
| 4815257-1 | 25 | 50 | (0) + | (GCTTT)n | Simple_repeat | 165 |
| 5023830-1 | 17 | 45 | (5) + | (TCCCT)n | Simple_repeat | 166 |
| 5148823-1 | 23 | 50 | (0) + | (TGGTT)n | Simple_repeat | 167 |
| 5245153-1 | 24 | 50 | (0) + | (TGGTT)n | Simple_repeat | 168 |
| 5604527-1 | 9 | 35 | (15) + | (CTTTC)n | Simple_repeat | 169 |
| 57505-21 | 26 | 50 | (0) + | (GTTTG)n | Simple_repeat | 170 |
| 5791730-1 | 25 | 50 | (0) + | (TGGTT)n | Simple_repeat | 171 |
| 5813231-1 | 15 | 48 | (2) + | (TTTCG)n | Simple_repeat | 172 |
| 6027129-1 | 26 | 50 | (0) + | (GTTTG)n | Simple_repeat | 173 |
| 615843-3 | 26 | 50 | (0) + | (GGTTT)n | Simple_repeat | 174 |
| 6170394-1 | 11 | 43 | (7) + | A-rich | Low_complexity | 175 |
| 647951-3 | 25 | 50 | (0) + | (GTTTG)n | Simple_repeat | 176 |
| 647951-3_1 | 25 | 50 | (0) + | (GTTTG)n | Simple_repeat | 177 |
| 6530453-1 | 24 | 49 | (1) + | (AACCA)n | Simple_repeat | 178 |
| 6532778-1 | 22 | 50 | (0) + | (TTGGG)n | Simple_repeat | 179 |
| 6919592-1 | 5 | 32 | (18) + | (ATTAA)n | Simple_repeat | 180 |
| 6936508-1 | 22 | 50 | (0) + | (TCCTT)n | Simple_repeat | 181 |
| 6997041-1 | 4 | 39 | (11) + | (GGCCGG)n | Simple_repeat | 182 |
| 7101384-1 | 25 | 50 | (0) + | (CGGTT)n | Simple_repeat | 183 |
| 71030-18 | 25 | 50 | (0) + | (TGGTT)n | Simple_repeat | 184 |
| 7138218-1 | 20 | 50 | (0) + | (TTGGT)n | Simple_repeat | 185 |
| 7277813-1 | 9 | 42 | (8) + | (TTCAG)n | Simple_repeat | 186 |
| 7438648-1 | 8 | 50 | (0) + | (CGTTG)n | Simple_repeat | 187 |
| 7561642-1 | 20 | 45 | (5) + | (TTTAT)n | Simple_repeat | 188 |
| 766555-2 | 21 | 50 | (0) + | (TTTGG)n | Simple_repeat | 189 |
| 7719774-1 | 25 | 49 | (1) + | (GTTGG)n | Simple_repeat | 190 |
| 8259080-1 | 12 | 42 | (8) + | (GGGAT)n | Simple_repeat | 191 |
| 8271026-1 | 25 | 50 | (0) + | (CGGTT)n | Simple_repeat | 192 |
| 8343365-1 | 24 | 50 | (0) + | (GCAGG)n | Simple_repeat | 193 |
| 8651868-1 | 26 | 50 | (0) + | (GGTTT)n | Simple_repeat | 194 |
| 8762139-1 | 24 | 50 | (0) + | (GGGCC)n | Simple_repeat | 195 |
| 9078830-1 | 9 | 34 | (16) + | (GTATT)n | Simple_repeat | 196 |
| 9135959-1 | 10 | 35 | (15) + | (CGTCG)n | Simple_repeat | 197 |
| 916488-2 | 25 | 50 | (0) + | (TGGTT)n | Simple_repeat | 198 |
| 9341664-1 | 22 | 48 | (2) + | (TTGGG)n | Simple_repeat | 199 |
| 9603049-1 | 13 | 48 | (2) + | (CGTAT)n | Simple_repeat | 200 |
| 9811864-1 | 23 | 50 | (0) + | (GTGCT)n | Simple_repeat | 201 |
| 9848076-1 | 21 | 48 | (2) + | (TTTGG)n | Simple_repeat | 202 |
| 9848076-1_1 | 21 | 48 | (2) + | (TTTGG)n | Simple_repeat | 203 |

**References**

1. Wales, N., Romero-Navarro, J. A., Cappellini, E. & Gilbert, M. T. P. Choosing the Best Plant for the Job: A Cost-Effective Assay to Prescreen Ancient Plant Remains Destined for Shotgun Sequencing. *PLoS One* **7,** e45644 (2012).

2. Wales, N., Andersen, K., Cappellini, E., Avila-Arcos, M. C. & Gilbert, M. T. P. Optimization of DNA recovery and amplification from non-carbonized archaeobotanical remains. *PLoS One* **9,** e86827 (2014).

3. Dabney, J. *et al.* Complete mitochondrial genome sequence of a Middle Pleistocene cave bear reconstructed from ultrashort DNA fragments. *Proc. Natl. Acad. Sci.* **110,** 15758–15763 (2013).

4. Schroeder, H. *et al.* Genome-wide ancestry of 17th-century enslaved Africans from the Caribbean. *Proc. Natl. Acad. Sci.* **112,** 3669–3673 (2015).

5. Meyer, M. & Kircher, M. Illumina sequencing library preparation for highly multiplexed target capture and sequencing. *Cold Spring Harb. Protoc.* **5,** (2010).

6. Wales, N. *et al.* New insights on single-stranded versus double-stranded DNA library preparation for ancient DNA. *Biotechniques* **59,** 368–371 (2015).

7. Close, T. J. *et al.* Development and implementation of high-throughput SNP genotyping in barley. *BMC Genomics* **10,** 582 (2009).

8. Da Fonseca, R. R. *et al.* The origin and evolution of maize in the American Southwest. *Nat. Plants Lett.* **1,** 1–5 (2015).

9. Bunning, S. L., Jones, G. & Brown, T. A. Next generation sequencing of DNA in 3300-year-old charred cereal grains. *J. Archaeol. Sci.* **39,** 2780–2784 (2012).
